# Supplementary material for: Discrepancies in Antimicrobial Susceptibility between the JP2 and the Non-JP2 Genotype of Aggregatibacter actinomycetemcomitans
Source: Antibiotics (Basel). 2022 Feb 27;11(3):317. doi: 10.3390/antibiotics11030317 (PMC8944592; doi:10.3390/antibiotics11030317)
Supplement: Supplementary file 1 [file antibiotics-11-00317-s001.zip › antibiotics-1586476-supplementary.pdf]

**Table S1.**

**The 160 strains *A. actinomycetemcomitans* strains used in the present work.** In total, 32 JP2 strains (1-32), 41 Ghanaian (Gh) (33-73), and 47 Swedish (Sw) serotype b strains, including strain J33 (74-120). In addition, 10 serotype a, and 10 serotype c strains from both Ghana and Sweden (120-160).

Cau, Caucasian descent, af, African descent

| Serial number | Strain number used in this study | Isolate number in strain collections | Origin of strains |
|---|---|---|---|
| 1 | J 1 | HK 921 | USA |
| 2 | J 2 | HK 1651 | Gh |
| 3 | J 3 | HK 1519 | Cape Verde |
| 4 | J 4 | HK 1702 | Brazil |
| 5 | J 5 | HK 909 | unknown |
| 6 | J 6 | KLA1-08 | Sw - cau |
| 7 | J 7 | BLA1-08 | Sw - cau |
| 8 | J 8 | 520-01 | Sw - cau |
| 9 | J 9 | 246 A-04 | Sw- af |
| 10 | J 10 | G24 | Gh |
| 11 | J 11 | G36 | Gh |
| 12 | J 12 | LA 640 | USA |
| 13 | J 13 | LA 806 | USA |
| 14 | J 14 | 090-10 | Sw - cau |
| 15 | J 15 | 196A1-10 | Sw - cau |
| 16 | J 16 | 437 G | Gh |
| 17 | J 17 | 488 G | Gh |
| 18 | J 18 | 524 G | Gh |
| 19 | J 19 | 654 G | Gh |
| 20 | J 20 | 666 G | Gh |
| 21 | J 21 | 3364 CV | Cape Verde |
| 22 | J 22 | 115A-11 | Sw - cau |
| 23 | J 23 | 352B-11 | Sw - cau |
| 24 | J 24 | 855G-11 | Gh |

| | | | |
|---|---|---|---|
| 25 | J 25 | 841G-11 | Gh |
| 26 | J 26 | 809G-11 | Gh |
| 27 | J 27 | 245-11 | Sw - cau |
| 28 | J 28 | P48 | Cape Verde |
| 29 | J 29 | 557 A-12 | Sw-af |
| 30 | J 30 | 338 A-13 | Sw - cau |
| 31 | J 31 | 342 A-13 | Sw-af |
| 32 | J 32 | 408 A-13 | Sw-af |
| 33 | 001 Ghb | 062 A | Gh |
| 34 | 028 Ghb | 197 A | Gh |
| 35 | 065 Ghb | 283 A | Gh |
| 36 | 071 Ghb | 286 A | Gh |
| 37 | 153 Ghb | 394 A | Gh |
| 38 | 205 Ghb | 367 A | Gh |
| 39 | 212 Ghb | 448 A | Gh |
| 40 | 217 Ghb | 457 A | Gh |
| 41 | 234 Ghb | 035 A | Gh |
| 42 | 369 Ghb | 180 A | Gh |
| 43 | 370 Ghb | 182 C | Gh |
| 44 | 443 Ghb | 001 A | Gh |
| 45 | 467 Ghb | 063 A | Gh |
| 46 | 470 Ghb | 067 A | Gh |
| 47 | 486 Ghb | 083 A | Gh |
| 48 | 490 Ghb | 096 A | Gh |
| 49 | 492 Ghb | 100 A | Gh |
| 50 | 493 Ghb | 136 A | Gh |
| 51 | 519 Ghb | 158 A | Gh |
| 52 | 540 Ghb | 186 A | Gh |
| 53 | 575 Ghb | 204 B | Gh |
| 54 | 581 Ghb | 208 A | Gh |
| 55 | 585 Ghb | 220 A | Gh |
| 56 | 589 Ghb | 284 A | Gh |
| 57 | 605 Ghb | 299 A | Gh |

| | | | |
|---|---|---|---|
| 58 | 619 Ghb | 372 A | Gh |
| 59 | 621 Ghb | 374 A | Gh |
| 60 | 624 Ghb | 376 A | Gh |
| 61 | 633 Ghb | 392 A | Gh |
| 62 | 638 Ghb | 222 A | Gh |
| 63 | 640 Ghb | 223 A | Gh |
| 64 | 652 Ghb | 412 A | Gh |
| 65 | 662 Ghb | 432 A | Gh |
| 66 | 670 Ghb | 423 A | Gh |
| 67 | 683 Ghb | 492 A | Gh |
| 68 | 702 Ghb | 439 A | Gh |
| 69 | 708 Ghb | 221 A | Gh |
| 70 | 716 Ghb | 448 A | Gh |
| 71 | 727 Ghb | 169 A | Gh |
| 72 | 744 Ghb | 326 A | Gh |
| 73 | 769 Ghb | 485 A | Gh |
| | | | |
| 74 | 11 Ub | 908-2000 | Sw |
| 75 | 12 Ub | 612-2002 | Sw |
| 76 | 14 Ub | 114-2005 | Sw |
| 77 | 27 Ub | 699-2000 | Sw |
| 78 | 40 Ub | 547-2004 | Sw |
| 79 | 54 Ub | 760-2003 | Sw |
| 80 | 66 Ub | 762-2002 | Sw |
| 81 | 78 Ub | 496-2002 | Sw |
| 82 | 107 Ub | 731-2002 | Sw |
| 83 | 112 Ub | 704-2001 | Sw |
| 84 | 119 Ub | 96-2000 | Sw |
| 85 | 128 Ub | 132-2002 | Sw |
| 86 | 135 Ub | 780-2003 | Sw |
| 87 | 137 Ub | 700-2001 | Sw |
| 88 | 139 Ub | 179-2001 | Sw |
| 89 | 145 Ub | 96-2005 | Sw |
| 90 | 146 Ub | 54-2006 | Sw |

| | | | |
|---|---|---|---|
| 91 | 147 Ub | 235-2006 | Sw |
| 92 | 148 Ub | 70-2008 | Sw |
| 93 | 153 Ub | 746-2002 | Sw |
| 94 | 160 Ub | 700-2002 | Sw |
| 95 | 167 Ub | 287-2009 | Sw |
| 96 | 172 Ub | 380-2009 | Sw |
| 97 | 177 Ub | 582-2009 | Sw |
| 98 | 184 Ub | 17-2010 | Sw |
| 99 | 185 Ub | 38-2010 | Sw |
| 100 | 189 Ub | 135-2010 | Sw |
| 101 | 195 Ub | 214-2010 | Sw |
| 102 | 196 Ub | 248-2010 | Sw |
| 103 | 197 Ub | 249-2010 | Sw |
| 104 | 200 Ub | 364-2010 | Sw |
| 105 | 201 Ub | 490-2010 | Sw |
| 106 | 208 Ub | 73-2011 | Sw |
| 107 | 219 Ub | 349-2011 | Sw |
| 108 | 231 Ub | 576-2011 | Sw |
| 109 | 234 Ub | 629-2011 | Sw |
| 110 | 240 Ub | 71-2012 | Sw |
| 111 | 244 Ub | 115-2012 | Sw |
| 112 | 251 Ub | 298-2012 | Sw |
| 113 | 255 Ub | 499-2012 | Sw |
| 114 | 260 Ub | 542-2012 | Sw |
| 115 | 266 Ub | 576-2012 | Sw |
| 116 | 279 Ub | 508-2007 | Sw |
| 117 | 281 Ub | 233-2004 | Sw |
| 118 | 282 Ub | 77-2002 | Sw |
| 119 | 290 Ub | 336-2008 | Sw |
| 120 | J 33 | 456 A-13 | Sw |
| | | | |
| 121 | 14 Gha | 187 A | Gh |
| 122 | 35 Gha | 208 A | Gh |
| 123 | 61 Gha | 272 A | Gh |

| | | | |
|---|---|---|---|
| 124 | 63 Gha | 278 A | Gh |
| 125 | 79 Gha | 295 A | Gh |
| 126 | 88 Gha | 300 A | Gh |
| 127 | 117 Gha | 358 A | Gh |
| 128 | 123 Gha | 365 A | Gh |
| 129 | 131 Gha | 368 B | Gh |
| 130 | 135 Gha | 372 B | Gh |
| | | | |
| 131 | 3 Ghc | 128 A | Gh |
| 132 | 16 Ghc | 187 B | Gh |
| 133 | 27 Ghc | 195 A | Gh |
| 134 | 75 Ghc | 292 A | Gh |
| 135 | 86 Ghc | 299 B | Gh |
| 136 | 92 Ghc | 304 A | Gh |
| 137 | 98 Ghc | 323 A | Gh |
| 138 | 109 Ghc | 347 A | Gh |
| 139 | 121 Ghc | 363 B | Gh |
| 140 | 129 Ghc | 368 A | Gh |
| | | | |
| 141 | 1 Uc | 686-2001 | Sw |
| 142 | 3 Uc | 553-2002 | Sw |
| 143 | 4 Uc | 483-2003 | Sw |
| 144 | 5 Uc | 28-2012 | Sw |
| 145 | 9 Uc | 544-2005 | Sw |
| 146 | 16 Uc | 375-2002 | Sw |
| 147 | 17 Uc | 35-2004 | Sw |
| 148 | 22 Uc | 276-2004 | Sw |
| 149 | 24 Uc | 37-2003 | Sw |
| 150 | 31 Uc | 141-2002 | Sw |
| | | | |
| 151 | 7 Ua | 522-2005 | Sw |
| 152 | 8 Ua | 22-2004 | Sw |
| 153 | 13 Ua | 178-2001 | Sw |
| 154 | 18 Ua | 400-2005 | Sw |

| | | | |
|---|---|---|---|
| 155 | 19 Ua | 648-2001 | Sw |
| 156 | 21 Ua | 371-2005 | Sw |
| 157 | 26 Ua | 182-2007 | Sw |
| 158 | 28 Ua | 650-2003 | Sw |
| 159 | 30 Ua | 659-2001 | Sw |
| 160 | 33 Ua | 549-2003 | Sw |

**Table S2. Comparison of MIC values for *A. actinomycetemcomitans* from different studies**.

MIC data given as two-fold dilutions

| Antimicrobial agent | MIC range | $MIC_{50}$ | $MIC_{90}$ | Number of strains, [reference] |
|---|---|---|---|---|
| **Amoxicillin** | 0.125-2.0 | 0.5 | 1 | 73, [25] |
| | 0.064-16 | 0.5 | 1 | 40 [27] |
| | 0.25-2 | 1 | 2 | 24 [23] |
| | 0.064-8 | 0.25 | 2 | 50 [22] |
| | 0.25-2 | 0.25 | 2 | 257 [28] |
| | 0.25-2 | 0.5 | 1 | 160 [present] |
| **Benzylpenicillin** | 0.5-8 | 2 | 4 | 73 [25] |
| | 0.125-8 | 2 | 8 | 50 [22] |
| | 0.125-8 | 1 | 4 | 160 [present] |
| **Azithromycin** | 0.064-2 | 1 | 1 | 73 [25] |
| | <0.064->32 | 0.5 | 32 | 24 [23] |
| | 0.125-64 | 4 | 16 | 40 [27] |
| | 0.125 - 4 | 2 | 4 | 45 [24] |
| | 0.064 - 4 | 0.5 | 1 | 160 [present] |
| **Cefotaxime** | 0.5-8 | 0.5 | 2 | 40 [27] |
| | <0.016 - 0.25 | 0.064 | 0.064 | 160 [present] |
| **Tetracycline** | 0.25-4 | 0.5 | 1 | 52 [25]* |
| | 0.25-1 | 1 | 1 | 21 [25] |
| | 0.032-16 | 0.25 | 0.5 | 50 [22] |
| | 0.125-16 | 2 | 8 | 40 [27] |
| | <0.125 -1 | 0.5 | 1 | 160 [present] |
| **Quinolones** | | | | |
| Moxifloxacin | 0.064-4 | 0.125 | 0.5 | 40 [27 |
| | 0.0064-0.032 | 0.0023 | 0.032 | 45 [24] |
| Trovafloxacin | ≤0.64-0.12 | ≤0.064 | ≤0.064 | 73 [25] |
| Ciprofloxacin | 0.01-0.08 | 0.03 | 0.04 | 50 [22] |
| | <0.002-0.006 | 0.03 | 0.006 | 45 [24] |
| Levofloxacin | <0.002 - 0.16 | 0.004 | 0.008 | 160 [present] |
| **Metronidazole** | 64->256 | >256 | >256 | 40 [27] |
| | 0.25->64 | 24 | >64 | 24 [23] |
| | 32->512 | 64 | 128 | 50 [22] |
| | 0.25-128 | 16 | 16 | 73 [25] |
| | 2- >256 | 8 | >256 | 45 [24] |
| | 0.5 - >256 | 4 | 128 | 160 [present] |

* Non-oral strains

**Table S3. The relationship between MIC values for benzylpenicillin (BP) and metronidazole in 160 strains of *Aggregatibacter actinomycetemcomitans*.**

| MIC BP (mg/L) | MIC metronidazole (mg/L) | | | | | | | | |
|---|---|---|---|---|---|---|---|---|---|
| | 1 | 2 | 4 | 8 | 16 | 32 | 64 | 128 | ≥256 |
| **8** | | 1 | | 1 | 1 | 3 | 2 | 2 | 1 |
| **4** | 1 | 1 | 3 | 7 | 5 | | | | 4 |
| **2** | 2 | 6 | 12 | 12 | 7 | 4 | 3 | 1 | 2 |
| **1** | | 2 | 8 | 5 | 5 | 5 | 1 | 1 | 3 |
| **0.5** | | 1 | 1 | 2 | 5 | 1 | 1 | | |
| **0.25** | 2 | 5 | 9 | 3 | 4 | 2 | | | |
| **0.125** | 1 | 6 | 4 | | | | | | |

The number of strains with respective MIC values is shown in the table.
